# Effects of Daily Vitamin D Supplementation on Objectively Measured Physical Activity: Results From the STURDY Trial

**DOI:** 10.1093/geroni/igaa057.2740

**Published:** 2020-12-16

**Authors:** Jennifer Schrack, Jacek Urbanek, Amal Wanigatunga, Stephen Juraschek, Christine Mitchell, Erin Michos, David Roth, Lawrence Appel

**Affiliations:** 1 Johns Hopkins Bloomberg School of Public Health, Baltimore, Maryland, United States; 2 Johns Hopkins University, Baltimore, Maryland, United States; 3 Harvard Medical School, Boston, Massachusetts, United States; 4 Johns Hopkins School of Medicine, Baltimore, Maryland, United States; 5 Johns Hopkins University School of Medicine, Baltimore, Maryland, United States

## Abstract

Cross-sectional evidence suggests older adults with higher serum vitamin D are more physically active, but whether long-term vitamin D supplementation attenuates age-related declines in physical activity (PA) is undefined. We examined the association between vitamin D supplementation and daily PA in 639 STURDY participants (aged 77 (5.4) years; 44% women) over up to 24-months. Participants were randomized to receive 200 (n=275), 1000 (n=168), 2000 (n=59), or 4000 (n=63) IU/day of vitamin D3. PA was measured using the Actigraph Link wrist-worn accelerometer 24 hours/day for 7-days at baseline, 3, 12, and 24 months. In linear mixed models adjusted for baseline PA level, total daily PA appeared to decline (β=-43.3 counts, p=0.06) annually for all groups and there was no difference by vitamin D3 dose (p for group*time =0.14). These results suggest daily vitamin D supplementation has no effect on quantities of daily PA.

